# Catatonia in a case of major depression resistant to pharmacotherapy. A case report

**DOI:** 10.1192/j.eurpsy.2023.1752

**Published:** 2023-07-19

**Authors:** C. M. Gil Sánchez, P. Casado de la Torre, M. V. Taillefer Aguanell

**Affiliations:** 1 Psychiatry, Sistema Andaluz de Salud (SAS), ALGECIRAS; 2 Psychiatry, Sistema Andaluz de Salud (SAS), Estepona, Spain

## Abstract

**Introduction:**

Catatonia is a clinical syndrome characterized by behavioral alterations, which may include motor immobility or excitation. As a symptom, catatonia may be present in several mental disorders, primarily schizophrenia and mood disorders. Symptoms can be severe and can lead to dangerous and lethal conditions if not diagnosed and treated properly.

**Objectives:**

To describe the complicated evolution of a case of major depression with psychotic symptoms, which developed catatonic status. We discuss the psychopharmacological approach and non-pharmacological therapies (ECT).

**Methods:**

Case summary. We have conducted a systematic review of the descriptions published to date, regarding this case.

**Results:**

We present a case of extreme severity, in a 55-year-old male, with a broad differential diagnosis with organic pathology, which required multidisciplinary management in conjunction with other specialties and multiple complementary tests.

Eventually diagnosed with major depression with psychotic symptoms evolving into a catatonic state. During more than one year of follow-up, multiple drugs have been tested sequentially: SSRI antidepressants, dual action, low-dose antipsychotics (caripracin, lurasidone, aripiprazole, olanzapine).

Finally, a good response was obtained in the treatment with lorazepam 1mg /6h and 12 sessions of ECT administered concomitantly.

In this case, the patient presented a refusal to eat and weight loss with a BMI of malnutrition. We had to be coordinated with the endocrinology service for a nutritional restitution strategy through dietary supplements. Once nutritional restitution was achieved, we started treatment with clomipramine, with good results on affective symptoms.

**Conclusions:**

Nowadays, the origin and treatment of catatonia are still unclear.

We present the case of a man with melancholic depression with psychotic symptoms, who evolved into a catatonic syndrome. A good response was achieved with the combination of ECT and benzodiazepines.

We want to highlight nutritional recovery as an important point to achieve good absorption of antidepressant drugs. Once achieved, we started treatment with clomipramine with good results.

During the treatment, he has presented multiple difficulties and finally, he was able to leave after five months of hospitalization in the acute mental health unit.

**Disclosure of Interest:**

None Declared

